# An Efficient PCR-RFLP Method for the Rapid Identification of Korean *Pyropia* Species

**DOI:** 10.3390/molecules22122182

**Published:** 2017-12-08

**Authors:** Yonguk Kim, Sung-Je Choi, Chulyung Choi

**Affiliations:** 1 Jeonnam Institute of Natural Resources Research, Jangheung-gun, Jeollanamdo 59338, Korea; kyu9801@hanmail.net; 2 Ocean & Fisheries Science Institute Haenam Branch, Haenam-gun 59046, Jeollanamdo, Korea; csjchoi@korea.kr

**Keywords:** *Pyropia*, PCR-RFLP, *rbcL*, *rps11–trnG*

## Abstract

The present study utilizes polymerase chain reaction-restriction fragment length polymorphism (PCR-RFLP) analysis using partial plastid *rbcL* and mitochondrial *trnC–trnP* gene sequences to distinguish the six representative *Pyropia* species produced via mariculture in Korea. The *rbcL*, *trnC*, and *trnP* sequences of 15 *Pyropia* species from the NCBI database were aligned to determine specific restriction enzyme sites of the six *Pyropia* species. To confirm the presence of restriction sites of eight enzymes, PCR amplicons were digested as follows: a 556 bp fragment within the *rbcL* region of chloroplast DNA was confirmed in *P. yezoensis* using *Bgl*I, whereas *Tth*111I, *Ava*II, *Bsr*I, and *Bsa*AI enzymes produced fragments of 664, 271, 600, and 510 bp, respectively, from the *rps11–trnG* region of mitochondrial DNA in *P. seriata*, *P. dentata*, *P. suborbiculata*, and *P. haitanensis*. In the case of *P. pseudolinearis*, *Hind*III, *Sac*II, and *Sph*I enzymes each had two cleavage sites, at positions 174 and 825, 788 and 211, and 397 and 602 bp, respectively. All six species were successfully distinguished using these eight restriction enzymes. Therefore, we propose that PCR-RFLP analysis is an efficient tool for the potential use of distinguishing between the six *Pyropia* species cultivated via mariculture in Korea.

## 1. Introduction

Red algae (Bangiales, Rhodophyta) of the genus *Pyropia*, including more than 75 species, are economically important mariculture crops and are available in China, Japan, and South Korea [1]. This genus is widely used as a dried sheet product, “zicai” in China, “nori” in Japan, and “gim” in South Korea, and is commonly consumed as a food source (laver) in Asian countries [2]. *Pyropia* is becoming increasingly popular worldwide. The economic value of *Pyropia* products exported in 2016 from South Korea was 353.3 million in US dollars [3,4]. Twelve species within the genus *Pyropia* have been recorded in South Korea [5]. Among them, four species, *P. yezoensis*, *P. seriata*, *P. dentata*, and *P. suborbiculata,* are widely distributed and extensively cultured in the South Jeolla Province, whereas *P. pseudolinearis* is distributed in Ulreung Island and its export accounts for about 80% of the total production of *Pyropia* in South Korea [3]. *P. haitanensis* is widely distributed and extensively cultured in the Fujian, Zhejiang, and Guangdong Provinces of China, and its export accounts for about 75% of the total production of *Pyropia* in China [6]. Recently, *P. haitanensis* has been utilized in Korean mariculture industries to develop improved cultivars that are optimized for the warm temperature zones typically used in *Pyropia* breeding.

The polymerase chain reaction-restriction fragment length polymorphism (PCR-RFLP) method is based on the digestion of PCR amplicons with appropriate restriction enzymes to produce distinct polymorphic fragments used as markers for species identification [7,8]. This method is beginning to be used for identifying different *Pyropia* species or breeding cultivars that could presumably be applied for mariculture in warming ocean waters. Japanese researchers have revealed clear differences between native *Pyropia* species based on PCR-RFLP analysis of plastid, internal transcribed spacer (ITS), and mitochondrial DNA (mtDNA) [9,10,11]. Chinese and Korean researchers have mainly used this method to identify and discriminate among seaweed diseases and pest species [12,13]. In South Korea, *Pyropia* species are identified using morphological and anatomical features. However, morphological identification of *Pyropia* taxa is limited because of the temporal variation in morphological characteristics and surface contamination from symbiotic seaweed and bacteria. Therefore, a better method is required for efficient and rapid identification to distinguish among these *Pyropia* species in natural environments.

In this study, we utilized PCR-RFLP analysis to successfully develop species-specific markers for five *Pyropia* species collected around the south-west coastal regions of South Korea, and *P. haitanenesis* collected from sites in China.

## 2. Results and Discussion

**Figure 1:**
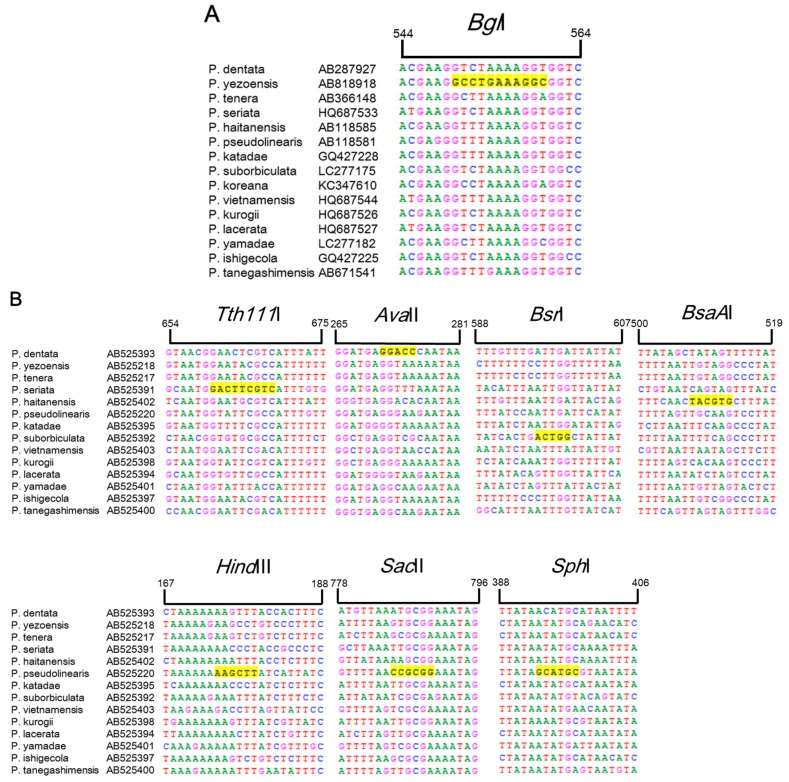
Sequence alignment of *rbcL* (**A**) and *rps11*–*trnG* (**B**) regions from 15 *Pyropia* species obtained from GenBank (National Center for Biotechnology Information (NCBI), Rockville, MD, USA). The bases highlighted in yellow correspond to the *Tth*111I, *Ava*II, *Bsr*I, *Bsa*AI, *Hind*III, *Sac*II, and *Sph*I enzyme restriction sites.

Currently, identification and discrimination between *Pyropia* species is primarily conducted based on classical taxonomic methods using microscopic analysis, which is limited by a lack of sufficient morphological features, time constraints, and inconsistencies between different taxonomists in South Korea [5]. Many phylogenetic analyses in other families have been based on the *trnC*–*trnP* region of mtDNA and the plastid *rbcL* intergenic spacer, as these two regions have high rates of variation between species [14,15]. Based on the partial sequences of *rbcL* and *trnC*–*trnP* for 15 *Pyropia* species available in the GenBank public database (National Center for Biotechnology Information (NCBI), Rockville, MD, USA), we found that six restriction enzymes were predicted to show species-specific RFLP patterns and could be used to identify the six *Pyropia* species using *rbcL* in *P. yezoensis* and *rps11*–*sdh3* in *P. seriata*, *P. pseudolinearis*, *P. dentate*, *P. suborbiculata*, and *P. haitanensis*. As shown in Figure 1, the predicted fragment sizes for the six *Pyropia* species were as follows: *Bgl*I produced a 556 bp fragment from *rbcL* of chloroplast DNA in *P. yezoensis*, whereas *Tth*111I produced a 664 bp fragment from *P. seriata*, *Ava*II produced a 271 bp fragment from *P. dentata*, *Bsr*I produced a 600 bp fragment from *P. suborbiculata*, and *Bsa*AI produced a 510 bp fragment from *P. haitanensis* in the *rps11*–*trnG* region of mtDNA. In the case of *P. pseudolinearis*, *Hind*III, *Sac*I, and *Sph*I produced one cleavage site in *rps11*–*trnG* at positions 174, 788 and 397 bp, respectively.

**Figure 2:**
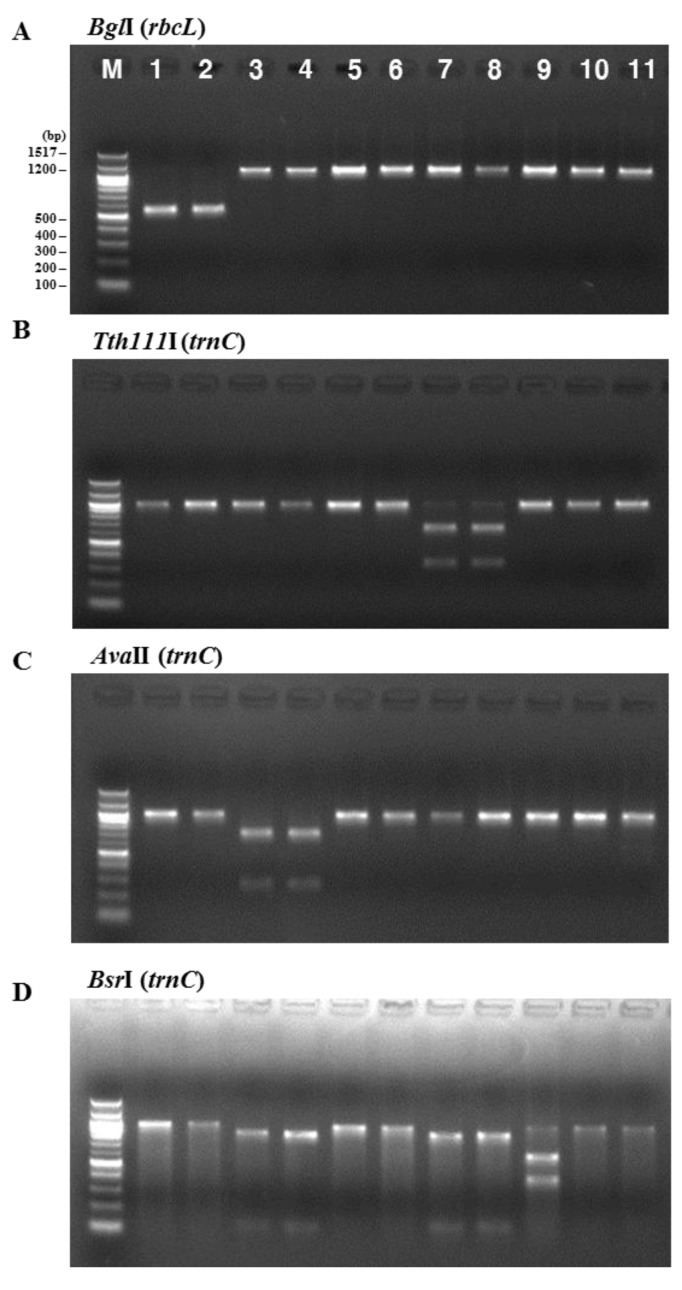

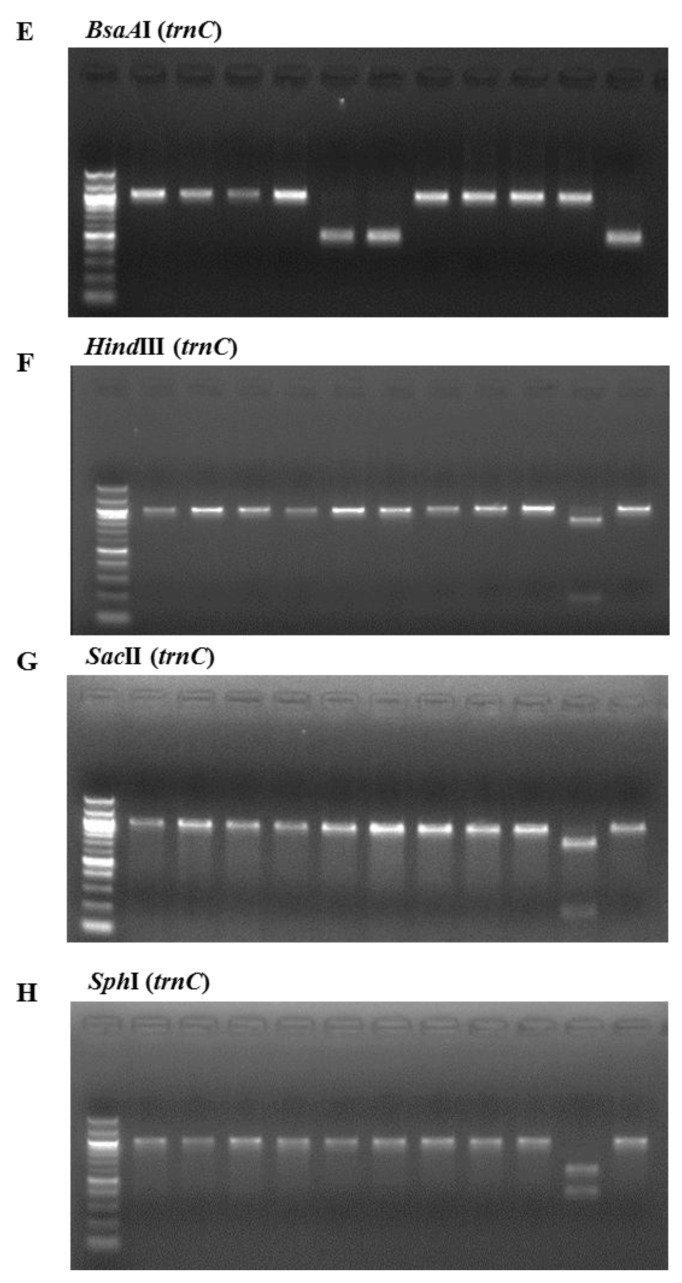
PCR-RFLP profiles of (**A**) the partial region of *rbcL* from all tested species digested with *Bgl*I; and the partial region of mitochondrial DNA including *trnC*–*trnP* digested with (**B**) *Tth*111I; (**C**) *Ava*II; (**D**) *Bsr*I; (**E**) *Bsa*AI; (**F**) *Hind*III; (**G**) *Sac*II; and (**H**) *Sph*I in *P. yezoensis*, *P. seriata*, *P. dentata*, *P. suborbiculata*, *P. haitanensis*, and *P. pseudolinearis*. Numbers indicate *Pyropia* samples as described in the Table 2.

The consistency of the presence of each enzyme restriction site was confirmed by analyzing PCR-RFLPs of 11 *Pyropia* samples collected at coastal areas in Korea and dried laver production in China. The samples were analyzed with the above restriction enzymes. Figure 2 illustrates the band sizes obtained by digestion using *Bgl*I for *rbcL* and *Tth*111I, *Ava*II, *Bsa*AI, *Bsr*I, *Hind*III, *Sac*I, and *Sph*I for *rps11*–*trnG*. *Bgl*I produced a 556 bp fragment in the *P. yezoensis* samples and was used for their identification (Figure 2A). The following enzymes were used to identify *P. seriata*, *P. dentata*, and *P. haitanensis*: *Tth*111I produced two fragments (664 bp and 333 bp) in *P. seriata* (Figure 2B), *Ava*II produced two fragments (271 bp and 732 bp) in *P. dentata* (Figure 2C), and *Bsa*AI produced one fragment (510 bp) in *P. haitanensis* (Figure 2E). *Bsr*I was used to identify *P. suborbiculata* samples and produced two fragments (600 bp and 401 bp) in this species, whereas *Bsr*I produced two fragments (119 bp and 844 bp) in both *P. seriata* and *P. dentata* (Figure 2D). Among four *Pyropia* samples that were origianlly identified as *P. dentata* using morphological and anatomical features, two samples were confirmed as *P. haitanensis* using our PCR-RFLP method (Figure 2E). We confirmed the ability of this PCR-RFLP assay to differentiate between *P. dentata* and *P. haitanensis*. Furthermore, three enzymes of *Hind*III, *Sac*II, and *Sph*I were used to identify *P. pseudolinearis* samples and produced two fragments of the 174 and 825 bp, 788 and 211 bp, and 397 and 602 bp, respectively (Figure 2F–H, Table 1).

The different restriction fragment patterns obtained for each species were analyzed to develop a discrimination method using PCR-RFLP analysis without the need for sequencing. Futhermore, our PCR-RFLP method showed that a higher sensitiviy and specificity to six *Pyropia* species found in Korean mariculture industires can be determined within a short time (approximately 2.5 h) compared to traditional morphological techiniques.

Most studies on PCR-RFLP analysis of *Pyropia* species have been conducted in Japan. Touhata et al. [16] demonstrated that the dried *Pyropia* products produed in China, Japan, and South Korea could be identified using PCR-RFLP analysis of the ITS-1 region. Abe et al. [9] distinguished 18 *Pyropia* species from Japan using restriction enzymes on mtDNA related to the *atp6* gene and partial mtDNA including the *trnC*, *rps11*, *sdh3*, *trnG*, *trnN*, *trnP*, and *rns* genes. However, these methods are hampered by the need for interpretation of complex RFLP patterns as well as the inter-laboratory differences and are relatively difficult to apply in the field.

The six species used in this study were successfully distinguished using a combination of eight restriction enzymes (*Bgl*I, *Tth*111I, *Ava*II, *Bsr*I, *Bsa*AI, *Hind*III, *Sac*II, and *Sph*I ) and two plastid and mitochodrial primer sets. These primer sets were designed based on the partial plastid and mtDNA sequences of 15 *Pyropia* species. As our study used only one or two samples per species, further studies should use a larger sample size as well as refined *Pyropia* markers based on a comparison with East Asian *Pyropia* species. 

## 3. Materials and Methods

### 3.1. Sequence Analysis

To rapidly identify the six *Pyropia* species, a PCR-RFLP method was designed base on the *rbcL* and *trnC*–*trnP* gene sequences from all the publicly available *Pyropia* species in the NCBI GenBank database (Table 2). These sequences were aligned by multiple sequence alignment (MSA) (Clustal Omega tool, EMBL-EBI, www.ebi.ac.uk) [17] and MEGA version 7.0 [18]. Scanning for the various restriction enzyme sites in the reference sequences was carried out using Restriction Mapper, version 3 (http://restrictionmapper.org). Restriction enzymes were selected based on the potential of showing variation between the reference sequences.

### 3.2. Sample Collection and DNA Isolation

Table 3 lists the six *Pyropia* species collected from China and South Korea used in this study. All samples were identified based on morphological characteristics. Cultures of the conchocelis stage of each specimen were maintained at the Ocean & Fisheries Science Institute, Haenam Branch, Jeollanamdo, Korea.

Prior to DNA extraction, conchocelis-stage samples of all strains (~300 mg wet weight) were washed twice with distilled water and frozen using liquid nitrogen, and then mechanically homogenized using a pestle tissue grinder. Genomic DNA was extracted from ground conchocelis-stage samples using the Qiagen DNeasy Plant Mini Kit (Qiagen, Valencia, CA, USA) according to the manufacturer’s instructions.

### 3.3. PCR Amplification and Restriction Digestion

PCR was performed using two 18S rDNA internal transcribed regions within *rbcL*; forward primer 5′-GCGAACGTTACGAATCTGGAGT-3′ and reverse primer 5′-ATGCTACTGGTACAACTTTACGTA-3′ (product size: 1107 bp), *trnC*–*P*; forward primer 5′-GTCAGTTCGAATCTGGCCCTAGTTT-3′ and reverse primer 5′-ATTCCCTTGCACCCAAAGCAAGTAC-3′ (approximately product size: 1005 bp). The PCR reactions were conducted in a 50 μL mixture containing 25 μL premix (Ex Taq Version 2.0, TaKaRa, Otsu, Japan), 50 ng genomic DNA templates, and 1 μL (10 pM) forward and reverse primers. The mixtures were denatured at 95 °C for 5 min and amplified with 40 cycles of 95 °C for 30 s, 55 °C for 20 s, and 72 °C for 30 s, with a final extension at 72 °C for 5 min. The PCR amplified products were electrophoresed on 1.5% agarose gels for 30 to 40 min.

Two μL of each PCR amplified *rbcL* and *trnC–P* product(concentration ranging from 0.6 to 1 μg/μL) was digested in 2 μL of 10× buffer, 1 unit of restriction enzyme, and 15.8 μL of distilled water in a final volume of 20 μL, followed by incubation at 37 °C for 1 h. The digested fragments were separated by electrophoresis on 1.5% agarose gels stained with ethidium bromide, and fragment patterns were visualized under UV light.

## Figures and Tables

**Table 1:** The fragment size (bps) for restriction digests of partial *rbcL* and *rps11*–*trnG* region in six *Pyropia* species.

	Restriction Enzymes							
Species	*Bgl*I	*Tth*111I	*Ava*II	*Bsr*I	*BsaA*I	*Hind*III	*Sac*II	*Sph*I
*P. yezoensis*	556	-	-	-	-	-	-	-
*P. seriata*	-	664 333	-	119 844	-	-	-	-
*P. dentata*	-	-	271 732	119 844	-	-	-	-
*P. suborbiculata*	-	-	-	600 401	-	-	-	-
*P. haitanenesis*	-	-	-	-	510	-	-	-
*P. pseudolinearis*	-	-	-	-	-	174 825	788 211	397 602

**Table 2:** DNA sequences of 15 *Pyropia* species used for RFLP analysis.

No.	Species	Source	Accession Number	
			*rbcL*	*trnC–trnP*
1	*P. dentata*	National Fisheries University, Shimonoseki, Japan	AB118579	AB525393
2	*P. yezoensis*	National Research Institute of Fisheries Science, Yokohama, Japan	AB818918	
		National Fisheries University, Shimonoseki, Japan		AB525218
3	*P. tenera*	Fisheries Technology Institute, Hyogo Prefectural Technology Center for Agriculture, Japan	AB366148	
		National Fisheries University, Shimonoseki, Japan		AB525217
4	*P. seriata*	Department of Biochemistry, University of Otago, New Zealand	HQ687533	
		National Fisheries University, Shimonoseki, Japan		AB525391
5	*P. haitanensis*	National Fisheries University, Shimonoseki, Japan	AB118585	AB525402
6	*P. pseudolinearis*	National Fisheries University, Shimonoseki, Japan	AB118581	AB525220
7	*P. katadae*	Department of Biology and Food Engineering, Changshu Institute of Technology, China	GQ427228	
		National Fisheries University, Shimonoseki, Japan		AB525395
8	*P. suborbiculata*	National Fisheries Research Institute, Nagasaki, Japan	LC277175	
		National Fisheries University, Shimonoseki, Japan		AB525392
9	*P. koreana*	Environmental Sciences, Botany, University of Girona, Girona, Spain	KC347610	
10	*P. vietnamesis*	Department of Biochemistry, University of Otago, New Zealand	HQ687544	
		National Fisheries University, Shimonoseki, Japan		AB525403
11	*P. kurogii*	Department of Biochemistry, University of Otago, New Zealand	HQ687526	
		National Fisheries University, Shimonoseki, Japan		AB525398
12	*P. lacerata*	Department of Biochemistry, University of Otago, New Zealand	HQ687527	
		National Fisheries University, Shimonoseki, Japan		AB525394
13	*P. yamadae*	National Fisheries Research Institute, Nagasaki, Japan	LC277182	
		National Fisheries University, Shimonoseki, Japan		AB525401
14	*P. ishigecola*	Department of Biology and Food Engineering, Changshu Institute of Technology, China	GQ427225	
		National Fisheries University, Shimonoseki, Japan		AB525397
15	*P. tanegashimensis*	National Fisheries Research Institute, Nagasaki, Japan	AB671541	
		National Fisheries University, Shimonoseki, Japan		AB525400

**Table 3:** *Pyropia* samples used in this study.

No.	Scientific Name	Common Name	Collection Site	Location
1	*P. yezoensis*	Bangsamunuigim	Songji-myeon, Haenam-gun, Jeollanam-do	34°21′05.92′′ N 126°27′40.76′′ E
2			Soan-myeon, Wando-gun, Jeollanam-do	34°08′53.12′′ N 126°41′10.12′′ E
3	*P. dentata*	Itbadidolgim	Yuldo-dong, Mokpo-si, Jeollanam-do	34°48′13.22′′ N 126°18′34.88′′ E
4			Palgeum-myeon, Sinan-gun, Jeollanam-do	34°46′13.04′′ N 126°10′22.44′′ E
5			Uisin-myeon, Jindo-gun, Jeollanam-do	34°19′07.66′′ N 126°17′31.87′′ E
6			Songji-myeon, Haenam-gun, Jeollanam-do	34°23′35.54′′ N 126°28′23.45′′ E
7	*P. seriata*	Momunuidolgim	Anjwa-myeon, Sinan-gun, Jeollanam-do	34°45′49.37′′ N 126°07′50.54′′ E
8			Songji-myeon, Haenam-gun, Jeollanam-do	34°45′49.37′′ N 126°07′50.54′′ E
9	*P. suborbiculata*	Dunggeundolgim	Nam-myeon, Yeosu-si, Jeollanam-do	34°25′32.73′′ N 127°47′31.33′′ E
10	*P. pseudolinearis*	Ginipdolgim	Ulleung-gun, Gyeongsangbuk-do	37°27′31.55′′ N 130°54′14.98′′ E
11	*P. haitanensis*	Haitanensisgim	Dried laver product from China	
